# Prevalence and Predictors of Low Birth Weight in a Rural Guatemalan Community

**DOI:** 10.26502/ogr073

**Published:** 2022-01-03

**Authors:** Emily S Himes, Claudia Rivera, Amy S Nacht, Saskia Bunge-Montes, Andrea Jimenez-Zambrano, Gretchen Heinrichs, Antonio Bolanos, Edwin Asturias, Stephen Berman, Margo S Harrison

**Affiliations:** 1 University of Colorado School of Public Health, Anschutz Medical Campus, Colorado, USA; 2 Fundación para la Salud Integral de los Guatemaltecos, Quetzaltenango, Guatemala (FSIG); 3 Denver Health, Colorado, USA

**Keywords:** Low Birth Weight, Prenatal Care, Maternal Age, Guatemala

## Abstract

**Background:**

The intention of our study was to establish the prevalence of low birth weight (LBW) as well as risk factors for LBW in infants born to a convenience sample of women enrolled in a home visitation maternal care program associated with the Center for Human Development in Southwest Trifinio, Guatemala.

**Methods:**

This is an observational study analyzing self-reported data from a quality improvement database. We recorded the distribution of birthweights of infants born to women enrolled in Madres Sanas that delivered between October 2018 and December 2019. We grouped women by LBW (<2500g ) and adequate birthweight (≥2500g) infants, and performed bivariate comparisons using sociodemographic, obstetric, and intrapartum data. Using the independent variables shown to have an association with LBW, we then performed a multivariable analysis.

**Results:**

There were 226 births among our program participants, 218 with recorded birthweights. The median birthweight was 3175g; 13.8% were LBW (<2500g), higher than Guatemala’s average of 10.9%. Through our bivariate analysis, we determined women with LBW infants were younger, with a median age of 20.8 (IQR [17.8-23.7]) compared to a median age of 23.2 (IQR [19.8-27.3]) among women with infants ≥2500g (*P*=0.03). Women with LBW infants were also more likely to have fewer than 4 prenatal visits (33.3% vs 19.3%, *P*=0.04).

**Conclusion:**

Two significant findings emerged from our analysis: LBW infants were more commonly born to women who were younger in age and who had received fewer than 4 prenatal visits. These findings are consistent with existing literature on LBW in Latin America. Our study helps to strengthen the data around these associations and gives credence to programming and policy efforts in Latin America that support adequate prenatal care for all and youth education about reproductive health and contraceptive access.

## 1. Introduction

The World Health Organization defines low birth weight (LBW) as less than 2500 grams at birth [1]. In 2015, the global prevalence of low birth weight was 14.6%, amounting to over 20 million newborns with inadequate weights [2]. Babies born under 2500g have increased risk of neonatal morbidity and mortality, and later on, developmental delays and chronic illness [1]. Considering the cost of illness and reduced work potential in adulthood, the impact of LBW can reach beyond a family’s health to their long-term social and economic well-being [3]. The World Health Assembly set a nutritional target for a 30% reduction in LBW between the years 2012 and 2025 [1]. In Latin America and the Caribbean, the rate of LBW was 8.8% in 2015, only slightly improved from 8.9% since 2000. Across the region the variations are pronounced, however: in 2015 Cuba’s rate of LBW was 5.3% in contrast to Guyana, which had a LBW rate of 15.6%; the prevalence in Guatemala was 10.9% [2]. Limited prenatal care has been associated with LBW in a number of studies based in Latin America [3, 4]. Prior studies have also found associations between LBW and educational level, being unmarried, living in a rural area [3], being at the extremes of reproductive age [3–6] and short interpregnancy intervals [4].

The intent in this study was to identify the prevalence of and risk factors associated with LBW in infants born to a convenience sample of women living in the lowlands of Southwestern Guatemala. Though this was a secondary analysis of a quality improvement database not designed to prospectively answer this question, we hypothesized that inadequate prenatal care, younger maternal age, and low levels of education would be associated with LBW in our convenience sample.

## 2. Methods

### 2.1 Setting

In 2011, a partnership between the University of Colorado and local agribusiness in the lowlands of Southwestern Guatemala resulted in the creation of the Center for Human Development (CHD) [7]. This organization has a clinic in the Southwest Trifinio region of Guatemala that houses outreach programming intended to improve maternal and child health outcomes in the area [7]. The Madres Sanas maternal health program is a community-based home visitation service delivered by specially trained nurses during the prenatal and postnatal periods [7]. It is currently designed to include 4 regular prenatal visits (PNVs), two postpartum visits, and additional unscheduled visits as needed [7]. The visits include physical assessment, screening for pregnancy complications, and referrals as needed [7]. The nurses also provide considerable education to women on themes such as danger signs of pregnancy, nutrition, breastfeeding, and contraceptive use. The nurses often utilize flipcharts and images to enhance content understanding [7]

### 2.2 Population/Methods

The population studied included a convenience sample of women enrolled in the Madres Sanas maternal health program. All enrolled women that delivered a live newborn between October 1, 2018 and December 3, 2019 were included in this study.

### 2.3 Ethics approval

This is a secondary analysis of a quality improvement database that has ethical approval from the Colorado Multiple Institutional Review Board (COMIRB # 15-0909). Given the quality improvement nature, women are not individually consented for collection of their data, but the data was anonymized.

### 2.4 Methods/Outcomes

This secondary data analysis of a quality improvement database has the primary outcome of LBW. LBW was defined as <2500 grams; data is collected by mother self-report of what she was told at the facility where she delivered or by the traditional birth attendant, although women sometimes present written documentation of birthweight. We compared the population of women with a LBW infant to those with a normal birthweight infant (≥2500 grams). We aimed to be able to identify sociodemographic (collected at the enrollment visit), obstetric (collected at enrollment), and intrapartum characteristics (collected at < 72 hour visit) associated with LBW. This data was collected by the community health nurses as part of the Madres Sanas program and was collected prospectively. It included demographic, antepartum, obstetric, and delivery characteristics.

### 2.5 Analysis

This analysis includes a description of the population of women observed during the study timeframe divided into the population of women who delivered a LBW infant compared to women with a normal weight infant. In bivariate comparisons we analyzed sociodemographic, antepartum, and intrapartum characteristics using Pearson’s chi-squared test for nominal categorical variables unless there was a low cell size, in which case Fisher’s exact test was used. The Kruskal-Wallis test was used for comparison of continuous variables. Variables with a *P*<0.05 were considered statistically significant, and then analyzed in a multivariable logistic regression. STATA software version 15.2 (StataCorp LP, College Station, TX, USA) was used for analysis.

## 3. Results

**Figure 1:**
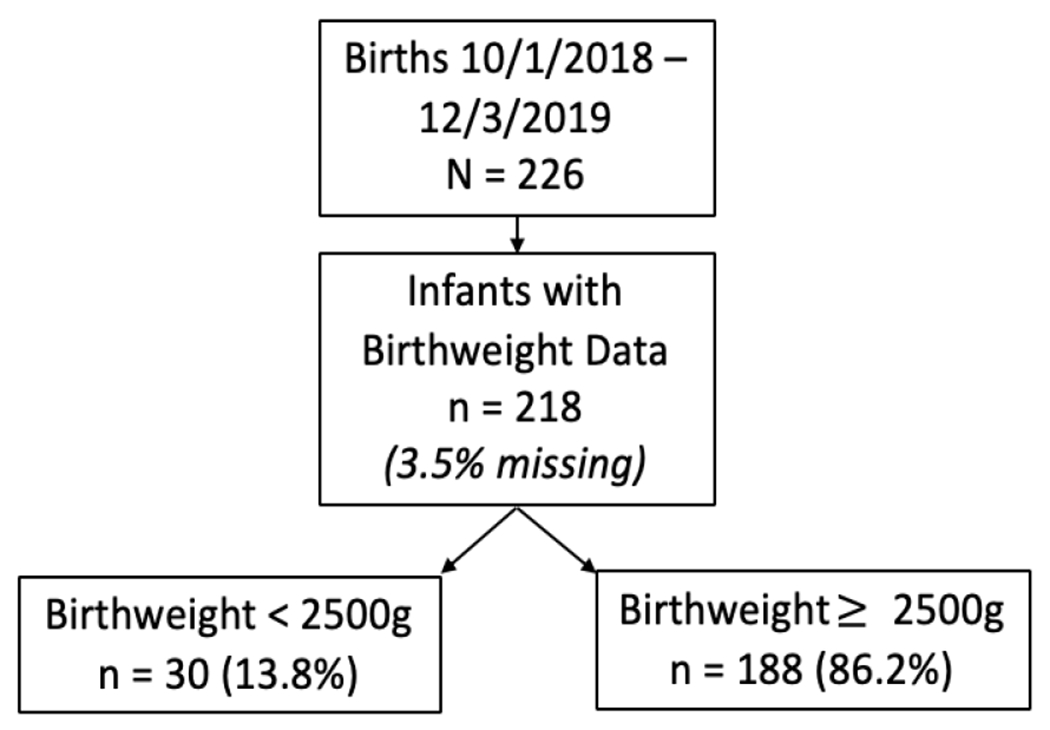
Study population by birthweight at birth.

**Figure 2:**
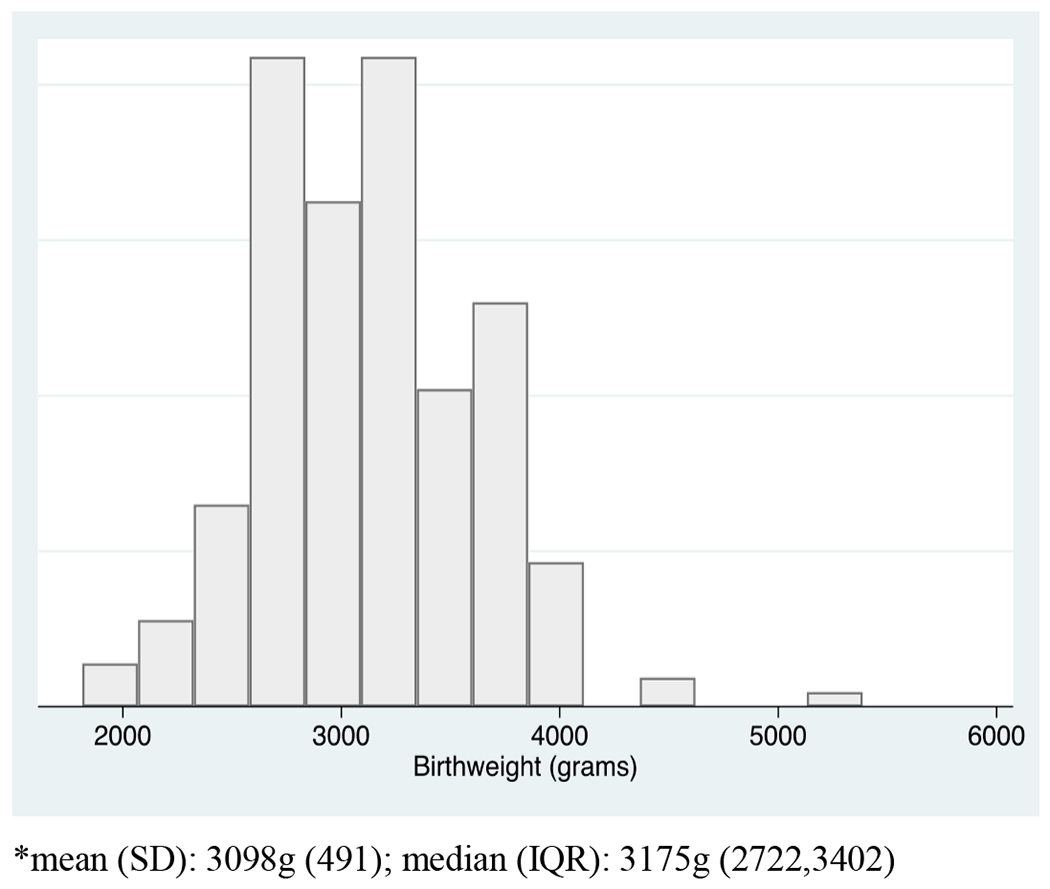
Distribution of Birthweight at Delivery in Convenience Sample.

Between October 1, 2018 and December 3, 2019 there were 226 live births in the Madres Sanas program. As shown in Figure 1, 218 of these births had a recorded birth weight, 30 (13.8%) were classified as LBW and 188 (86.2%) had an adequate birth weight. Figure 2 is a histogram showing the distribution of birth weights across the population. The median birthweight was 3098 grams with an interquartile range (IQR) of 2722 to 3402 grams. In Table 1, sociodemographic, antepartum, and obstetric characteristics are listed overall and across the comparison groups of interest in columns 3 though 5. Median age for the 218 women included in the study was 22.7 years (IQR [19.4-26.9]). 12.4% of women received no formal education with 63.6% attending school through the primary years (6^th^ grade); 12% of the population reported being illiterate. Most women were married (88.1%) with 1.9% of women reporting employment. The population included women from 12 communities in the area; 56% of women lived within close proximity to the CHD and 44% lived in areas considered to be far from the CHD. Communities designated as “far” are still reachable within 30 minutes on a motorcycle.

Regarding antepartum and obstetric characteristics, most women were multiparous (56.9%). Prior to conception, 23.7% reported using contraception. 80.7% of the women were seen at least 4 times during their pregnancy for antenatal care. The cesarean birth rate was 48.6% of all births, and 65.1% of women had a history of at least one prior cesarean birth. Home births occurred in 30.9% of the population, 69.1% of deliveries were in a hospital or clinic setting, and 74% had a skilled attendant at the birth (nurse or physician). In the bivariate analysis, women with LBW infants were younger, with a median age of 20.8 (IQR [17.8-23.7]) compared to a median age of 23.2 (IQR [19.8-27.3]) among women with infants ≥2500g (*P*=0.03). Women with LBW infants were also more likely to have fewer than 4 prenatal visits (33.3% vs 19.3%, *P*=0.04). The remaining sociodemographic and obstetric characteristics were not found to have a significant association with LBW in bivariate comparisons. After determining a significant association between both maternal age and number of prenatal visits and the outcome variable of LBW, we performed a logistic regression to examine the relationship further. In this multivariable analysis (Table 2), being a year older in age was protective against low birth weight (0R 0.9, 95% CI [0.8,0.9], *P*=0.03). Additionally, women with 4 or more prenatal visits were 60% less likely to have a LBW infant (0R 0.4, 95% CI [0.2-0.9], *P*=0.04).

## 4. Discussion

In this analysis we gained insight into the prevalence of and predictors associated with LBW in a convenience sample of women living in rural Southwest Guatemala. From our quality improvement database, we determined the prevalence of LBW infants born to women enrolled in the Center for Human Development’s Madres Sanas program was 13.8%, considerably higher than the national statistic of 10.9% reported by UNICEF in 2015 [2]. LBW infants were more frequently born to younger mothers (median age 20.8 vs 23.2, *P*=0.03). We also found LBW infants were 60% less likely to be born to women that had received more than 4 prenatal visits. Significant findings in the analysis help to inform ways in which the LBW rates could be reduced. The correlation we found between younger age and LBW is not a novel finding, as many other studies have endorsed an association between adolescent mothers and LBW in Latin America [4, 5]. Our results regarding LBW being more common in younger women confirm the importance of delaying pregnancy, especially when that confers other benefits on young women such as finishing school and fewer obstetric complications [6].

Additionally, it supports the need for education about and access to contraception for adolescents. The Center for Human Development has another program, “Big Decisions,” an initiative focused on sexual education and knowledge about contraception for youth in the community, which is intended to prevent pregnancy and could have implications for LBW. A number of Latin American studies have identified access to adequate prenatal care as a key factor in improving birth outcomes and preventing LBW, as we found in our analysis [3, 4]. There is, however, research demonstrating equivocal results regarding the efficacy of prenatal care [8]. These mixed results likely stem from the wide spectrum of prenatal care quality and the varied contexts within which it is provided. Our study population is comprised of women from a low socioeconomic status, rural community with low health literacy; therefore, it is unsurprising that they benefit from the prenatal care, education, and support provided by our nurses. The Madres Sanas program is a thoughtful and coordinated program that provides valuable care and education about warning signs, medical screening, physical assessment, and counseling on psychosocial issues as well as nutrition. Additionally, connecting the women to local health institutions for pregnancy complications and their deliveries helps to mitigate some of the risk associated with pregnancies in remote areas.

The limitations of this study include its small sample size, the use of self-reported data, the convenience sample study design, the secondary analysis study design, and the relatively short window of time in which data was collected. Additionally, several variables are notably absent from the analysis, namely validated measures of nutrition and gestateonal age. Maternal weight gain and anemia have been shown to be associated with LBW in prior research [10], and gestational age would have allowed for differentiation of prematurity from small for gestateonal age as a cause of LBW. While this information is currently being collected for the quality improvement database, there was considerable missing data in the timeframe of our study, therefore these variables were omitted for our analysis. Missing data related to pregnancy intervals required that we exclude this variable from analysis as well. In other studies, closely spaced pregnancies have been associated with increased risk of LBW [4], which is an important finding supporting increased contraceptive access in lower income countries where multiple pregnancies in quick succession may be more common.

## 5. Conclusion

In conclusion, analysis of our quality improvement database utilizing data obtained through the Madres Sanas program revealed a higher prevalence of LBW (13.8%) than is typically seen in Latin American countries [2, 3, 5]. LBW infants were more likely to be born to younger mothers and mothers that had received fewer than 4 prenatal visits, though we were unable to evaluate the association of important variables such as maternal weight gain, anemia, gestational age and pregnancy spacing.

## Figures and Tables

**Table 1:** Bivariate comparisons of the association of maternal sociodemographic and obstetric characteristics by low birth weight versus adequate birth weight.

Sociodemographic Characteristics	Total Population (n = 218)	LBW < 2500g (n=30, 13.8%)	Adequate BW ≥2500g (n=188, 86.2%)	P value
	**n = 216, 0.9% missing**			
**Age in years (Median, IQR)**	22.7 [19.4,26.9]	20.8 [17.8,23.7]	23.2 [19.8,27.3]	0.03^a^
**Education**	**n = 217, 0.5% missing**			0.77^b^
None	27 (12.4%)	4 (13.3%)	23 (12.2%)	
Primary	138 (63.6%)	17 (56.7%)	165(87.8%)	
Basic	37 (17.1%)	6 (20.0%)	31 (16.6%)	
Diversified	15 (6.9%)	3 (10.0%)	12 (6.4%)	
**Literacy**	**n = 217, 0.5% missing**			1.0^b^
Yes	191 (88.0%)	27 (90%)	164 (87.7%)	
No	26 (12.0%)	3 (10%)	23 (12.30%)	
**Employed**	**n = 215, 1.4% missing**			1.0^b^
Yes	4 (1.9%)	0 (0.0%)	4 (2.2%)	
No	211 (98.1%)	30 (100.0%)	181 (97.8%)	
**Single**	**n = 211, 3.2% missing**			1.0^b^
No (Married)	192 (88.1%)	27 (90.0%)	165 (87.8%)	
Yes	26 (11.9%)	3 (10.0%)	23 (12.2%)	
**Community**	**0% missing**			0.93
Far	96 (44.0%)	13 (43.3%)	83 (44.2%)	
Close	122 (56.0%)	17 (56.7%)	105 (55.9%)	
**Obstetric and Antepartum Characteristics**				
**Parity (at enrollment, prior to delivery)**	**0% missing**			0.67^b^
0	8 (3.7%)	0 (0.0%)	8 (4.3%)	
1	39 (17.9%)	7 (23.3%)	32 (17.0%)	
2	47 (21.6%)	7 (23.3%)	40 (21.3%)	
3+	124 (56.9%)	16 (53.3%)	108 (57.5%)	
**Took Birth Control Before Conception**	**0% missing**			0.26
Yes	51 (23.7%)	9 (32.1%)	42 (22.5%)	
No	164 (76.3%)	19 (67.9%)	145 (77.5%)	
**Number of Madres Sanas PNVs** *	**0% missing**			0.04
< 4	42 (19.3%)	10 (33.3%)	32 (17.0%)	
4+	176 (80.7%)	20 (66.7%)	156 (83.0%)	
**History of Prior Cesarean Birth**	**0% missing**			0.83
0	76 (34.9%)	11 (36.7%)	65 (34.6%)	
1+	142 (65.1%)	19 (63.3%)	123 (65.4%)	
**Delivery Characteristics**				
**Mode of Delivery**	**0% missing**			0.53
Vaginal Birth	112 (51.4%)	17 (56.7%)	95 (50.5%)	
Cesarean Birth	106 (48.6%)	13 (43.3%)	93 (49.5%)	
**Location of Delivery**	**n = 217, 0.5% missing**			0.98
Home or Other	67 (30.9%)	9 (31.0%)	58 (30.9%)	
Facility (Clinic or Hospital)	150 (69.1%)	20 (69.0%)	130 (69.2%)	
**Birth Attendant**	**n = 223, 1.3% missing**			0.8
Comadrona (TBA, “unskilled”)	56 (26.0%)	7 (24.1%)	49 (26.3%)	
Nurse or Physician (“skilled”)	159 (74.0%)	22 (75.9%)	137 (73.7%)	

* PNVs: prenatal visits, WHO: World Health Organization, TBA: traditional birth attendant

Note: *P* value is the result of chi-squared testing unless otherwise noted

a : Kruskal-Wallis test

b : Fisher’s exact test

**Table 2:** Multivariable Model of Characteristics Associated with Low Birthweight.

	Odds Ratio	95% CI	*P* value
Being a year older in maternal age	0.9	0.8, 0.9	0.03
Achieving 4 or more prenatal visits	0.4	0.2, 0.9	0.04

Note: all covariates with P<0.05 in bivariate comparisons were included in a logistic regression of association of the variable with low birthweight.
